# Robot-assisted stereotactic electroencephalography using 3-dimensional intraoperative imaging and frameless registration tool

**DOI:** 10.3171/2024.4.FOCVID2419

**Published:** 2024-07-01

**Authors:** Luca Paun, Alessandro Moiraghi, Giorgia Antonia Simboli, Johan Pallud, Marc Zanello

**Affiliations:** Departments of 1 Neurosurgery and; 4 Neuropathology, Groupe Hospitalier Universitaire Paris Psychiatrie et Neurosciences, Hôpital Sainte-Anne, Université Paris Cité, Paris, France;; 2 Institute of Psychiatry and Neuroscience of Paris, INSERM U1266, Paris, France; and; 3 Division of Neurosurgery, Department of Clinical Neurosciences, Geneva University Hospitals and University of Geneva Faculty of Medicine, Geneva, Switzerland

**Keywords:** epilepsy surgery, stereotactic technique, robotic surgical procedure, drug-resistant epilepsy, frameless stereotaxy

## Abstract

Stereoelectroencephalography (SEEG) is the gold standard to investigate the epileptic network in cases of drug-resistant epilepsy. Robot-assisted SEEG is increasingly being used; however, its installation process in the operating room is more difficult than that of the stereotactic frame procedure. New robotic tools and 3D intraoperative imaging ease the setup while achieving the same mechanical precision and a lower complication rate. In this video, the authors illustrate the surgical technique and step-by-step workflow using a robotic arm (neuromate) guided by a frameless registration system (neurolocate), registered with an intraoperative flat-panel CT scanner (O-arm).

The video can be found here: https://stream.cadmore.media/r10.3171/2024.4.FOCVID2419

## Transcript

### 0:25 Introduction and Surgical Indication.

Stereoelectroencephalography (SEEG) is the gold standard to investigate epileptogenic networks in drug-resistant epilepsy.^1^ This technique was introduced for the first time at Sainte-Anne Hospital on May 3, 1957, by Dr. Bancaud and Dr. Talairach^2–4^ and progressively adopted worldwide. Since its introduction in our institution, this procedure has been performed 1536 times. The aim of this surgical procedure is to record cortical activity thanks to deep electrodes implanted under stereotactic conditions, allowing to understand the implications of specific brain cortical zones in seizure onset and/or propagation. The SEEG procedure has numerous advantages that make it the gold standard: 1) the ability to record and monitor simultaneously multiple brain zones, even bilaterally or deep-seated; 2) the low morbidity due to a mini-invasive approach without craniotomy; 3) the potential use of implanted electrodes as a curative treatment via radiofrequency thermocoagulation.

At the beginning, SEEG’s main aim was to circumscribe an epileptogenic zone and to determine if this zone was accessible for surgical treatment via cortectomy. Due to the developments and refinements of the procedure, SEEG is nowadays used to investigate epileptogenic networks, searching to interrupt and/or disrupt epileptogenic aberrant loops.^5–7^

### 1:48 Preoperative Planning: Imaging and Trajectory Planning.

The software used to plan the trajectories is Brainlab Elements. Brainlab Elements is a surgical software allowing to perform image coregistration, DTI analysis, and trajectory planning. Nonenhanced T1-weighted 3D fast spin echo MRI is used as the reference series: vascular and functional images are coregistered to this reference series. The lead trajectories are defined as a straight line between one entry point (EP) and one target point (TP), in agreement with the surgical epileptologists during a preoperative multidisciplinary board meeting. The definition of the epileptogenic zone is based on preoperative noninvasive investigations: at first, results of video-EEG recordings; clinical examination with a complete neuropsychological assessment; then the imaging data with 3T, 7T, and functional MRI; and finally the metabolic imaging with a PET-MRI. This workup must lead to a first delimitation of a quite restricted possible seizure onset zone and spatiotemporal seizure propagation patterns: the electrodes’ trajectories are then placed into the corresponding anatomical structures keeping in mind its low spatial resolution. The trajectories are also planned with a 2-mm margin distance from at-risk zones (vessels and sulci) by two neurosurgeons, blind to each other. Vascular images, in particular 3D angiography sequences, are acquired for each patient and used to adjust the trajectories. The idea is to avoid, and lower as much as possible, any risk of hematoma or bleeding during or after the procedure. The trajectories are validated during a dual consensus. In our experience, a dozen electrodes per SEEG is sufficient to investigate a delimitated epileptic area: if the epileptogenic zone hypothesis is not supported by the SEEG analysis, a second SEEG procedure could be performed to investigate a second zone. The increase in number of implanted electrodes does not necessarily increase the identification of an epileptogenic zone and could complicate the understanding of the procedure by the patient: delimitate a well-defined area to perform a cortectomy. Once the definitive trajectories are validated, the Digital Imaging and Communications in Medicine (DICOM) coordinates of each trajectory are transferred to the neuromate (Renishaw) robot control station.

## Surgical Workflow

### 4:06 Patient Positioning and Registration.

Surgical setup consists in positioning the patient’s head in a Talairach head clamp (DIXI). The Talairach head clamp is still in use in Sainte-Anne Hospital, but the neuromate robot can be used with other head fixation systems such as the Leksell frame G.^8^ During this step, it is important to maintain the head in a neutral position and to include as much as possible the skull base in the O-arm (Medtronic) field of view: the aim is to ease the coregistration between 3D O-arm series acquired intraoperatively and those of the preoperative MRI. Positioning and intraoperative planning procedure is similar to the robot-assisted stereotactic biopsy performed in our institution.^8–10^ Artifacts due to the Talairach head clamp (or any other head fixation system) should be taken into consideration during intraoperative imaging acquisition. As for a classical CT scan, one profile and one anteroposterior x-ray (XR) images are acquired, to position the head at the center of the O-arm field of view. The final O-arm position is then registered.

### 5:05 Robot Calibration.

Before starting the surgical procedure, the robot is to be checked. On the robot control station, the NMControl program is switched on and a test procedure is started. During this phase, the accuracy of the robot, the system communication, the different reference positions, the anticollision system, and the remote control are verified. If the test is passed, the robot can be used.

### 5:33 Imaging Coregistration.

The laser tool holder is installed on the robotic arm, and it has to be firmly attached thanks to two fixation screws. The neurolocate (Renishaw) registration module is positioned on the laser tool holder: this is the frameless system consisting of 5 ruby spheres, enabling intraoperative registration without the need for bone or skin anchored fiducials. It is of utmost importance to have it correctly aligned because it will position the robot into the operative room space. The neurolocate registration tool must be positioned as close to the patient’s head as possible and aligned with the calvaria on the vertex, reducing the space between the skin and the neurolocate ruby spheres. A 3D O-arm acquisition is made and verified: all the 5 ruby spheres must be identified. The 3D acquisition is performed at a low-dose level to minimize patient irradiation. The image is coregistered to the preoperative MRI. The imaging coregistration is roughly made manually and then refined by automatic algorithm. The quality of the coregistration process is verified by two neurosurgeons and the planned trajectories are controlled again.

### 6:36 Second O-Arm Acquisition and Accuracy Control on Skin Surface.

An additional safety check is realized. The robot is positioned into one trajectory axis: a wire metallic pin inserted into the standard tool holder is put in contact with the patient’s skin. A 3D O-arm series is acquired, then coregistered with preoperative MRI: the O-arm metallic pin has to be precisely aligned with the planned trajectory. This allows a final evaluation of the robot accuracy.

### 7:01 Guiding Screw Installation.

The O-arm can be moved away from the surgical field, thanks to the registration of its position during the first step of the procedure. For each trajectory, the robotic arm is aligned on the trajectory axis. A minimal hair shaving is performed around the EP located on the skin. The robotic arm is then moved as close as possible to the patient’s head to minimize mechanical deviation during the drilling process. The drill hole is performed with a 2.5-mm-diameter drill bit: the drilling movement must be as smooth as possible and the bit as sharp as possible. The length of the drill bit is defined to perforate exclusively the bone without damaging the dura mater: a locking device is secured on the drill to execute this procedure with a minimal risk.

Once the bone hole is made, the durotomy is realized thanks to a thin monopolar coagulation. The guiding screw dimension is chosen according to a homemade software, SEEGapp, and then secured into the skull. The distance between the distal part of the guiding screw and the TP located into the brain parenchyma guides the choice of the electrode length and number of contacts. A stylet is introduced before the electrode: this preliminary trajectory minimizes trajectory deviation due to electrode’s flexibility. The electrode is slowly pushed into the parenchyma and fixed thanks to the guided screw. The procedure is repeated in the same manner for each electrode.

### 8:31 Intraoperative Control.

Once all the electrodes are positioned, a new 3D O-arm series is acquired using the registered O-arm position. The new image is fused to the preoperative MRI and the trajectories are compared. If any electrode presents an important deviation at the target point (≥ 2 mm), the corresponding electrode is removed, the length is adjusted, and then it is replaced. Another 3D O-arm series is performed. This procedure is repeated until all the trajectories respect the preoperative planification.

### 9:05 Park Position and End of the Procedure.

The robot is moved in park position. The electrodes’ cables are fastened to the scalp by surgical stitches. Lastly, the Talairach head clamp is removed starting from the anterior pins moving subsequently to the posterior ones.

## Acknowledgments

We thank Marc Harislur.

## Disclosures

The authors report no conflict of interest concerning the materials or methods used in this study or the findings specified in this publication.

## Author Contributions

Primary surgeon: Zanello. Assistant surgeon: Paun, Moiraghi. Editing and drafting the video and abstract: Zanello, Paun, Moiraghi, Simboli. Critically revising the work: all authors. Reviewed submitted version of the work: all authors. Approved the final version of the work on behalf of all authors: Zanello. Supervision: Zanello, Moiraghi, Pallud.

## Supplemental Information

### Patient Informed Consent

The necessary patient informed consent was obtained in this study.

## References

[1] Cardinale F Rizzi M Vignati E Stereoelectroencephalography: retrospective analysis of 742 procedures in a single centre Brain 2019 142 9 2688 2704 31305885 10.1093/brain/awz196

[2] Zanello M Roux A Duriez P Jean Talairach: the man behind the cerebral stereotactic space Brain 2022 145 2 411 415 34919651 10.1093/brain/awab463

[3] Talairach J Bancaud J Bonis A Szikla G Tournoux P Functional stereotaxic exploration of epilepsy Confin Neurol 1962 22 328 331 13984743 10.1159/000104378

[4] Bancaud J Angelergues R Bernouilli C Functional stereotaxic exploration (SEEG) of epilepsy Electroencephalogr Clin Neurophysiol 1970 28 1 85 86 4188481

[5] Isnard J Taussig D Bartolomei F French guidelines on stereoelectroencephalography (SEEG) Neurophysiol Clin 2018 48 1 5 13 29277357 10.1016/j.neucli.2017.11.005

[6] Cardinale F Casaceli G Raneri F Miller J Lo Russo G Implantation of stereoelectroencephalography electrodes: a systematic review J Clin Neurophysiol 2016 33 6 490 502 27918344 10.1097/WNP.0000000000000249

[7] Cardinale F Cossu M SEEG has the lowest rate of complications J Neurosurg 2015 122 2 475 477 10.3171/2014.7.JNS141680 25526269

[8] Zanello M Simboli GA Carron R Pallud J MRI-based and robot-assisted stereotactic biopsy with intraoperative CT imaging Acta Neurochir (Wien) 2022 164 12 3311 3315 35821282 10.1007/s00701-022-05271-1

[9] Deboeuf L Moiraghi A Debacker C Feasibility and accuracy of robot-assisted, stereotactic biopsy using 3-dimensional intraoperative imaging and frameless registration tool Neurosurgery 2023 92 4 803 811 36700740 10.1227/neu.0000000000002294

[10] Elia A Paun L Pallud J Zanello M Robot-assisted endoscopic third ventriculostomy under intraoperative CT imaging guidance Acta Neurochir (Wien) 2023 165 9 2525 2531 37488400 10.1007/s00701-023-05713-4 PMC10570216

